# Herceptin Enhances the Antitumor Effect of Natural Killer Cells on Breast Cancer Cells Expressing Human Epidermal Growth Factor Receptor-2

**DOI:** 10.3389/fimmu.2017.01426

**Published:** 2017-10-30

**Authors:** Xiao Tian, Feng Wei, Limei Wang, Wenwen Yu, Naining Zhang, Xinwei Zhang, Ying Han, Jinpu Yu, Xiubao Ren

**Affiliations:** ^1^Department of Immunology, Tianjin Medical University Cancer Institute and Hospital, Tianjin, China; ^2^Department of Biotherapy, Tianjin Medical University Cancer Institute and Hospital, Tianjin, China; ^3^National Clinical Research Center of Cancer, Tianjin, China; ^4^Key Laboratory of Cancer Immunology and Biotherapy, Tianjin, China; ^5^Cancer Molecular Diagnostic Center, Tianjin Medical University Cancer Institute and Hospital, Tianjin, China

**Keywords:** natural killer cells, breast cancer, Herceptin, antibody-dependent cell-mediated cytotoxicity, CD16

## Abstract

Optimal adoptive cell therapy (ACT) should contribute to effective cancer treatment. The unique ability of natural killer (NK) cells to kill cancer cells independent of major histocompatibility requirement makes them suitable as ACT tools. Herceptin, an antihuman epidermal growth factor receptor-2 (anti-HER2) monoclonal antibody, is used to treat HER2^+^ breast cancer. However, it has limited effectiveness and possible severe cardiotoxicity. Given that Herceptin may increase the cytotoxicity of lymphocytes, we explored the possible augmentation of NK cell cytotoxicity against HER2^+^ breast cancer cells by Herceptin. We demonstrated that Herceptin could interact with CD16 on NK cells to expand the cytotoxic NK (specifically, CD56^dim^) cell population. Additionally, Herceptin increased NK cell migration and cytotoxicity against HER2^+^ breast cancer cells. In a pilot study, Herceptin-treated NK cells shrunk lung nodular metastasis in a woman with HER2^+^ breast cancer who could not tolerate the cardiotoxic side effects of Herceptin. Our findings support the therapeutic potential of Herceptin-treated NK cells in patients with HER2^+^ and Herceptin-intolerant breast cancer.

## Introduction

Clinical studies have revealed the promising effects of adoptive cell therapy (ACT) in cancer treatment (1). The success of ACT requires the therapeutic strategies to meet four critical criteria, including clinical efforts that should focus on obtaining sufficient numbers of immune effector cells, the ability of the immune cells to reach the tumor site and recognize the targets, efficient tumor cell killing, and minimal or no harm caused to healthy cells. Increasing evidence has been reported to support the application of natural killer (NK) cells for ACT. NK cells, a component of innate immunity, are cytotoxic lymphocytes that play an important role in the host’s early protective responses against transformed cells (2). It has been reported that CD3^−^CD56^+^ NK cells can exert a main histocompatibility complex-independent antitumor effect on both solid and hematological malignancies without affecting hematopoietic precursors or normal tissues (3, 4). Furthermore, the results from a clinical study support the therapeutic efficacy of an NK cell infusion in patients with colon carcinoma (5).

Natural killer cells, a heterogeneous population of lymphocytes, represent a small fraction of the peripheral blood mononuclear cells (PBMCs) (6). Since NK cells are significant effectors of the innate immune system and have a unique ability to directly lyse transformed or virus-infected cells without prior sensitization or MHC class restriction, they are regarded as promising candidates for immunotherapy of cancer (7–9). However, breast cancer patients in all clinical stages of disease had significantly decreased NK cell activity and that it was most profound in advanced stage of this disease (10, 11). Sufficient numbers of fully functional NK cells are usually generated *ex vivo* to achieve satisfactory therapeutic efficacy. Currently, several clinical studies have focused upon adoptive autologous NK cells infusion in an attempt to treat common malignancies, such as breast cancer, lymphoma, renal cell carcinoma and non-small cell lung cancer (6, 12–14). Cytokines, such as interleukin (IL)-2, IL-7, IL-10, IL-15, or IL-18 have been reported to amplify NK cells isolated from PBMCs (15–17). Previous studies from our laboratory have shown that the combination of IL-2 and IL-15 stimulated the expansion of NK cells, without affecting the cytotoxicity of NK cells (18).

Breast cancer is the most commonly diagnosed cancer and is the second highest cause of cancer death in women (19). In China, breast cancer is the most common cancer for females (20). Herceptin is a widely used human epidermal growth factor receptor-2 (HER2)-targeted therapy for treating metastatic breast cancer by down-regulating tumor cell proliferation. Herceptin is an anti-HER2 monoclonal antibody, which is engineered by inserting the complementary determinant regions of a murine antibody (clone 4D5) into the consensus framework of human IgG1 (21). In addition to common side effects, such as fever, rash and infection, a severe side effect, cardiotoxicity, limits the application of Herceptin in some patients. Nakagawa et al. found that Herceptin could increase the cytotoxicity of lymphocytes and Herceptin-activated lymphocytes could inhibit the growth of breast cancer cells *in vitro* (22). Therefore, the aim of the present study was to identify the effects of engaging NK cells with Herceptin on the activities of NK cells under IL-2 and IL-15 stimulation conditions. We found that Herceptin increased the NK cell proliferation, migration, and cytotoxicity against HER2^+^ cancer cells. These results revealed a new function of Herceptin for increasing antitumor effects of NK cells, in addition to directly suppressing proliferation in HER^+^ cancer cells and support the application of targeted antibodies against tumor cells to enhance the clinical efficacy of ACT.

## Materials and Methods

### Generation and Characterization of NK Cells

This study was carried out in accordance with the recommendations of the ethical standards of the Institutional Review Committee on Human Research of the Tianjin Medical University Cancer Institute and Hospital with written informed consent from all subjects. All subjects gave written informed consent in accordance with the Declaration of Helsinki. The protocol was approved by the Institutional Review Committee on Human Research of the Tianjin Medical University Cancer Institute and Hospital.

Peripheral blood mononuclear cells were obtained from female patients who were pathologically diagnosed with breast cancer. The method of NK cell expansion was previously reported by our group (18). The day before Day 0, T75 flasks were separately treated with Herceptin at 1 mg/ml (Roche, Swiss), IgG1 at 1 mg/ml (Abcam, USA), or same volume of washed twice with phosphate-buffered saline (PBS). At Day 0, we pour out the coating liquid and washed flasks twice with PBS. On Day 0, PBMCs were isolated from enriched peripheral blood by Ficoll-Hypaque density gradient centrifugation, washed twice with PBS, and cultured in GT551-H3 serum free medium (TaKaRa Biomedical Technology, Japan) supplemented with 10% fetal bovine serum plus IL-2 (10 ng/ml) and IL-15 (50 ng/ml, Peprotech Inc., USA), in the presence or absence of pretreatment T75 flasks. The culture condition was a temperature of 37°C in the humidified atmosphere of a CO_2_ incubator. Cells cultured in the PBS-treated flasks were served as controls. The medium was changed every 3 days with the addition of GT551-H3 serum free medium supplemented with 10% fetal bovine serum plus IL-2 (10 ng/ml) and IL-15 (50 ng/ml). Expanded NK cells were transferred to T125 flasks at a density of 1.0 × 10^6^ cells/ml on Day 5. On Day 10, the cells were transferred to cell culture bags at 1.0 × 10^6^ cells/ml. Cells were harvested at Day 15 and enriched using a MACS^®^ human NK cell negative-selection isolation kit (Miltenyi Biotec, Germany) according to the manufacturer’s instructions. Briefly, non-target cells were indirectly magnetically labeled with a cocktail of biotin-conjugated antibodies against lineage-specific antigens and a cocktail of MicroBeads. The magnetically labeled non-target cells were depleted by retaining them on a MACS^®^ Column in the magnetic field of a MACS Separator, while the unlabeled NK cells passed through the column.

### Breast Cancer Cell Culture

A breast cancer cell line, SK-BR-3 cells, was cultured in RPMI 1640 (Takara Biomedical Technology, Japan) supplemented with 10% fetal bovine serum (Thermo Fisher Scientific, USA), penicillin (100 IU/ml; Sigma, USA), and streptomycin (100 µg/ml; Sigma, USA).

### Small-Interfering RNA (siRNA) Transfection

The siRNA technique was employed to silence the expression of the HER2 gene of SK-BR-3 cells. Double-stranded siRNAs were transfected into SK-BR-3 cells using Lipofectamine 2000 (Thermo Fisher Scientific, USA) according to the manufacturer’s instructions. The siRNA sequences used to silence the HER2 gene were 5′-GCAGUUACCAGUGCCAAUATT-3′ (sense) and 5′-UAUUGGCACUGGUAACUGCTT-3′ (antisense). The non-targeting sequences, 5′-UUCUCCGAACGUGUCACGUTT-3′ (sense) and 5′-ACGUGACACGUUCGGAGAATT-3′ (antisense), were used as the negative control.

### Flow Cytometry (FCM) Analysis

Cells from cultures for 0, 5, 10, and 15 days were labeled using following PE-, FITC-, APC-, or PerCP-conjugated mouse antihuman antibodies in fluorescence activated cell sorter buffer (BD Biosciences, USA) for 30 min at room temperature; the antibodies included anti-CD16 (3G8), anti-NKp30 (P30-15), anti-NKG2D (1D11), anti-DNAM-1 (11A8), and anti-CD107a (H4A3) (Biolegend, USA) as well as CD3-FITC and CD56-PE (BD Biosciences). APC- and FITC-conjugated and purified antimouse IgG1 (Biolegend) were used as isotype controls. Goat antihuman IgG (Thermo Fisher Scientific) and the matched isotype control antibody were used to stain cells and to identify cells binding with Herceptin and IgG. Cells were analyzed using FACS Calibur and FACS CantoII instruments (BD Biosciences, USA) and FlowJo software to determine the percentage of positive cells in CD3^−^CD56^+^ NK cell populations.

### Cytotoxicity Assay

The cytotoxicity of NK cells was measured using the CytoTox 96 non-radioactive cytotoxicity assay kit (Promega, USA) according to the manufacturer’s instructions. Briefly, enriched NK cells (4 × 10^6^/ml) were cultured with the same volume of SK-BR-3 or SK-BR-3/HER2si cells (0.25 × 10^5^/ml, 0.5 × 10^5^/ml, and 1 × 10^5^/ml) in a 96-well plate for 4 h at 37°C in a humidified CO_2_ incubator prior to removal of the supernatant for lactate dehydrogenase (LDH) release measurements. LDH release is previously standardized to represent the percentage of cytotoxicity using this formula (23, 24):

$$\begin{array}{l} \%\text{Cytotoxicity}=\text{(Experimental-Effector spontaneous-Target spontaneous)}/\\ \;\left(\text{Target maximum-Target spontaneous}\right)\times 100. \end{array}$$

### CD107a Degranulation Assay

Enriched NK cells (4 × 10^6^/ml) were cultured with the same volume of SK-BR-3 or SK-BR-3/HER2si cells (1 × 10^5^/ml) for 4 h at 37°C in a humidified CO_2_ incubator. Monensin (Sigma, USA) was added at a final concentration of 2 µl/ml after 1 h of incubation. Cells were then stained for CD107a and CD3/CD56/CD45. The percentage of CD107a-positive cells was estimated using FCM by gating for CD3^−^CD56^+^ NK cells. Spontaneous CD107a expression was detected in the absence of target cells.

### Enzyme-Linked Immunosorbent Assay (ELISA)

Enriched NK cells (8 × 10^6^/ml) were cultured with the same volume of SK-BR-3 or SK-BR-3/HER2si cells (2 × 10^5^/ml) for 24 h at 37°C in a humidified CO_2_ incubator. The concentrations of interferon (IFN)-γ in cell culture supernatants obtained from three independent experiments were detected using commercial ELISA kits (DAKEWE, China), according to the manufacturer’s instructions. The human IFN-γ kit sensitivity was 5 pg/ml.

### Migration and Adhesion of NK Cells to Target Cells

Target cells, SK-BR-3 cells, were labeled with carboxyfluorescein succinimidyl amino ester using a Cell Labeling Kit (Abcam) according to the manufacturer’s instructions. Effector cells (NK cells) and labeled target cells were mixed at an effector: target ratio of 40:1 in a final volume of 500 µl of GT551-H3 medium without IL-2 in a 35 mm glass-bottom cell culture dish. The cells were allowed to settle at the bottom of the dish for 2 min. Images were immediately captured every 30 s for up to 2 h using a 40× magnification objective on an Andor Revolution living-cell imaging station (Olympus) while cells were maintained at 37°C. The images in the figures were excised from live-cell imaging videos at different time points. Finally, twenty target cells from each group were randomly chosen and the cumulative number of adherent NK cells was from 20-min periods up to 2 h. The average number of adherent NK cells in each group was determined.

### Statistical Analysis

The statistical significance of the differences between two groups was determined with a two-tailed, paired Student’s *t*-test. Differences among multiple groups were analyzed with one-way analysis of variance using Prism 6 software (GraphPad). Values are expressed as the medians or means ± SD, as indicated, and differences were considered statistically significant at *p* < 0.05.

## Results

### Herceptin Increased NK Cell Proliferation

**Figure 1:**
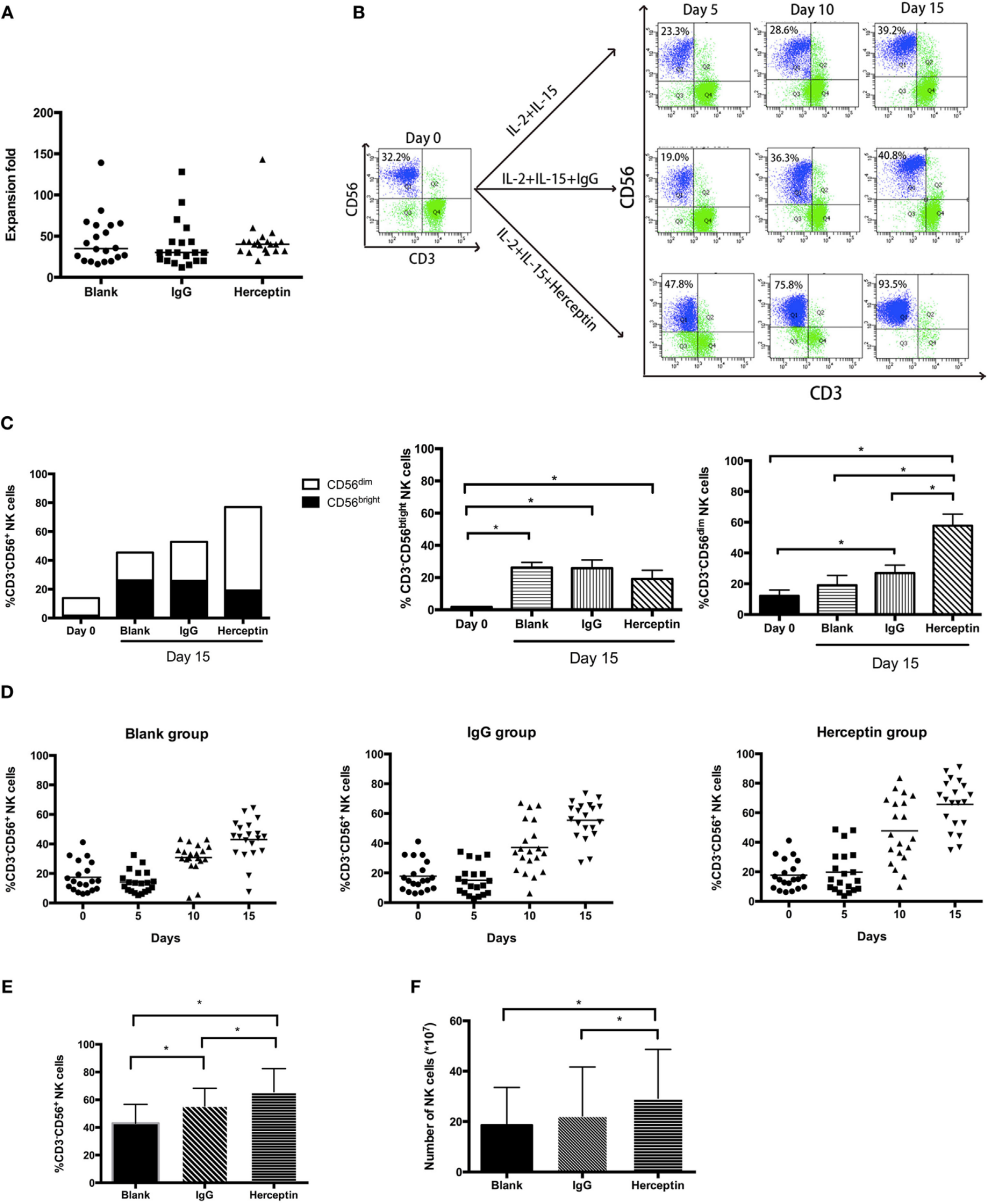
The expansion of *ex vivo* expanded human natural killer (NK) cells in the presence of interleukin (IL)-2 and IL-15. Freshly prepared peripheral blood mononuclear cells (PBMCs) were cultured in flasks coated with IgG1 or Herceptin for 15 days. **(A)** Expansion fold of the final *ex vivo* expanded products. We counted the cell number on Day 15 and determined the expansion fold. The results are presented as the median values for 20 patients. **(B)** A typical change in NK cell expansion observed among 20 samples under different culture conditions. The percentage of NK cells (CD56^+^CD3^−^) in the expanded PBMCs culture was determined by flow cytometry (FCM). The left panel represents the primary PBMCs. Rows of the right nine graphs from top to bottom represent the blank group, the IgG1 group and the Herceptin group; columns from left to right represent the expansion products obtained after 5, 10, and 15 days. **(C)** The percentages of CD56^bright^ and CD56^dim^ subsets of NK cells. We evaluated the NK cell subsets in three samples from the three groups on Days 0 and 15. **(D)** Changes in the percentage of NK cells over 15 days under different culture conditions. The graphs from left to right show the blank group, the IgG1 group and the Herceptin group. **(E)** Percentages of NK cells in the final expansion products of three groups. **(F)** The number of NK cells in the three groups observed on Day 15. The results shown in **(E,F)** are presented as the means of 20 patients. The results are presented as the means of independent experiments, and the error bars represent the SD. *p < 0.05.

We first investigated the effects of Herceptin on NK cell proliferation by evaluating the percentage and number of NK cells treated with IL-2 plus IL-15 (blank group), as well as IL-2/IL-15 in combination with IgG1 (IgG1-treated group) and Herceptin (Herceptin-treated group) at various time periods. During 15 days culture, we measured cell number of three groups every 5 days. The final product was a 40-fold enrichment (range, 16–139-fold) after culture for 15 days in Herceptin-treated group, which was similar to that in the blank and IgG1-treated group (Figure 1A). The gating strategy used to determine the percentages of CD56^dim^ and CD56^bright^ NK cells is shown in Figure S1 in Supplementary Material. The typical changes in the percentage of NK cells in these 3 groups by using NK cells from 20 patients were evaluated (Figure 1B). At Day 0, we observed 2 groups of primary NK cells, CD56^bright^ NK cells and CD56^dim^ NK cells. Based on these findings, Herceptin, IgG1, and cytokines amplified the CD56^bright^ NK cells. Cells in IgG1 and blank groups were maintained as 2 populations until Day 15. Notably, in the Herceptin-treated group, most of the amplified NK cells observed on Day 15 were CD56^dim^ cells (57.80 ± 7.60%) compared with Day 0; however, but in the Blank-NK group, most of the amplified NK cells were CD56^bright^ cells (26.23 ± 3.25%) (Figure 1C). The overall trends in the changes in the percentages of NK cells are shown in Figure 1D. All three groups showed increases in the percentages of NK cells after 15 days, but Herceptin-treated NK cells had the greatest proliferation during this period (from 17.68 ± 9.94 to 65.71 ± 16.72%) (Figure 1E). When we calculated the number (the number of final cells multiplied by the percentage of NK cells) of NK cells at Day 15, we found that Herceptin efficiently increased the number of NK cells (29.26 ± 19.38 × 10^7^), compared with the IgG1 group (22.22 ± 19.47 × 10^7^) and blank group (18.57 ± 14.99 × 10^7^) (Figure 1F).

Therefore, Herceptin increases the percentage of NK cells in the final expansion products.

### Herceptin Increased the Cytotoxicity of NK Cells against HER2^+^ Breast Cancer Cells

**Figure 2:**
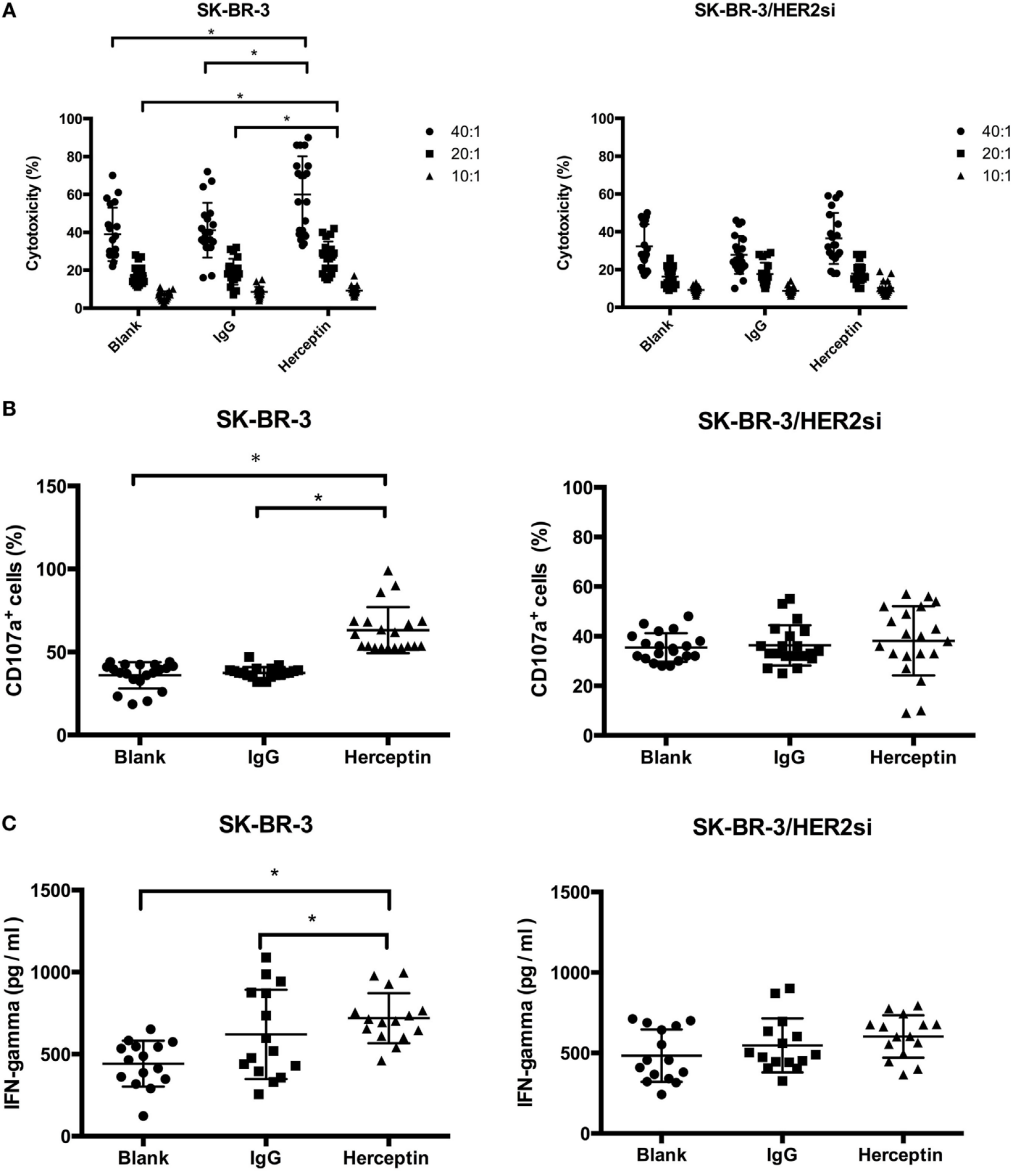
Cytotoxicity of expanded natural killer (NK) cells toward breast cancer cells. For the cytotoxicity assay, NK cells were enriched prior to the assay. **(A)** The cytotoxicity of expanded NK cells toward SK-BR-3 and SK-BR-3/HER2si cells was measured using the lactate dehydrogenase (LDH) release assay at 40:1, 20:1, 10:1 effector-to-target (E:T) ratios for 4 h. **(B)** NK cell degranulation after coculture with cancer cells. The cytotoxicity of expanded NK cells toward SK-BR-3 and SK-BR-3/HER2si cells was measured using a CD107a release assay at 40:1 effector-to-target (E:T) ratios. Differences in the frequency of CD107a^+^ cells among NK cells in the Herceptin, IgG1 and blank groups were analyzed for statistical significance. **(C)** The level of interferon (IFN)-γ secretion after coculture with cancer cells. NK cells were stimulated with the indicated targets, SK-BR-3 and SK-BR-3/HER2si cells. After 3 days, supernatants were collected and IFN-γ levels were measured. The results are presented as the means of 15 patients and error bars represent the SD. *p < 0.05.

Next, we evaluated the cytotoxicity of NK cells using three approaches, including LDH release, target cell degranulation measured using CD107a expression, and IFN-γ secretion. Herceptin-treated NK cells had higher levels of cytotoxicity toward HER2^+^ cells compared to blank and IgG1 treated groups when the E:T ratio was 40:1 or 20:1; however, Herceptin-treated NK cells did not display higher cytotoxicity than IgG1 treated and blank groups for HER2^−^ cells (Figure 2A). Herceptin-treated NK cells also increased the expression level of CD107a on the cell surface in HER2^+^ cells, but not HER2^−^ cells, suggesting that degranulation is increased when target cells are engaged with HER2^+^ cells (Figure 2B). Consistent with the results obtained from the LDH and CD107a release assays, Herceptin-treated NK cells induced a higher level of IFN-γ secretion when cocultured with HER2^+^ cells but not with HER2^−^ cells (Figure 2C). These results indicate that Herceptin treatment increases the NK cell cytotoxicity on HER2^+^ breast cancer cells. This effect may be caused by increasing NK cell activity toward cancer cells and direct effects of Herceptin on cancer cells.

### Herceptin Improved the Migration and Adhesion of NK Cells to HER2^+^ Breast Cancer Cells

**Figure 3:**
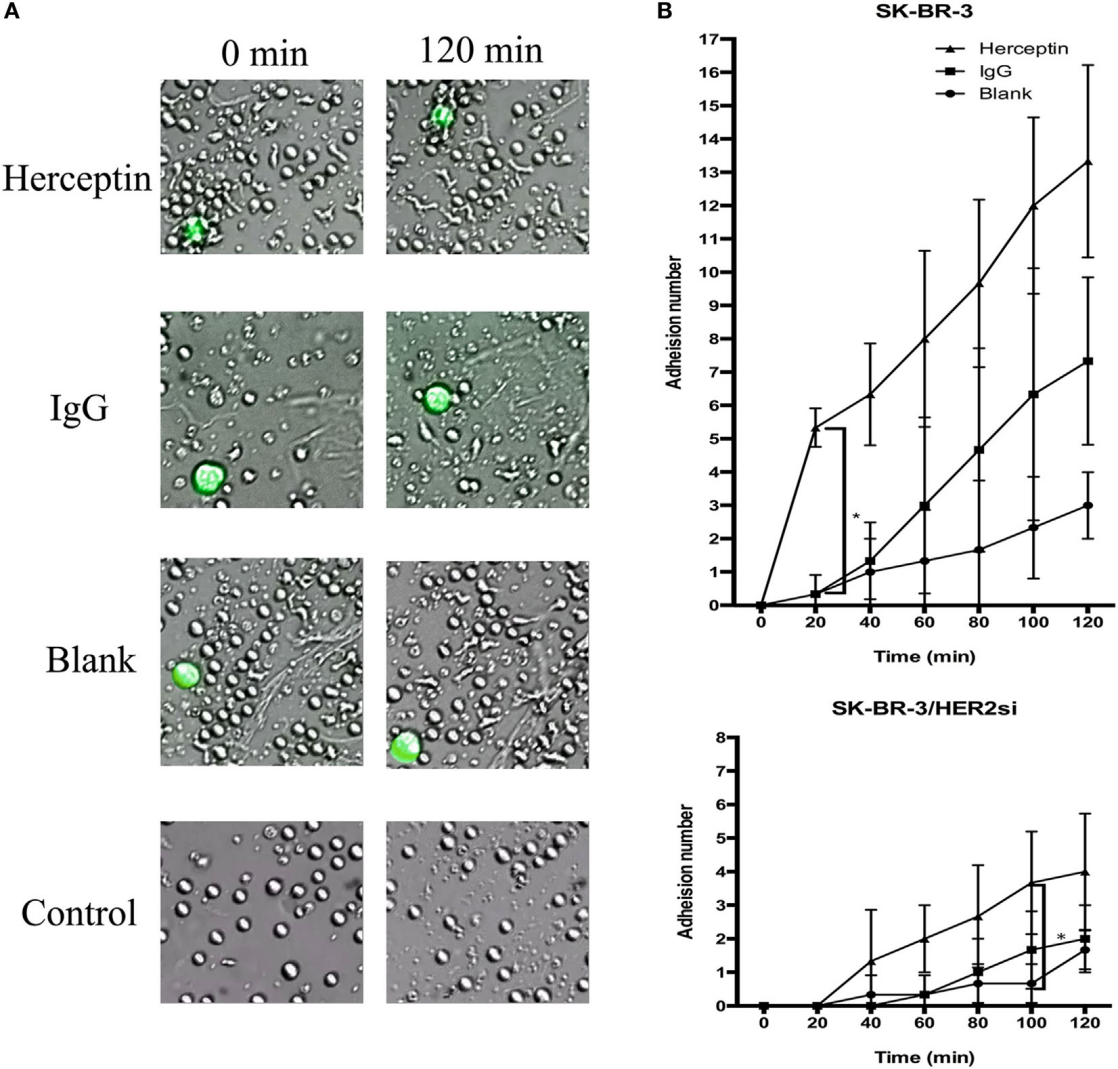
The migration and adhesion of expanded natural killer (NK) cells. The larger green cells in the photos are target cells and the smaller cells are NK cells. **(A)** Images of expanded NK cells cocultured with SK-BR-3 at 0 min (left row) and 120 min (right row). **(B)** Statistical analysis of the calculated number of adherent NK cells. The results are presented the means of three independent experiments and error bars represent the SD. *p < 0.05.

To examine the effects of Herceptin treatment on NK cell migration and adhesion to breast cancer cells, NK cells were cocultured with HER2^+^ and HER2^−^ cells in the live-cell imaging station for 2 h. A greater frequency of Herceptin-treated NK cells migrated faster toward SK-BR-3 cells (Video S1 in Supplementary Material), and NK cells in the blank group were not changed (Video S2 in Supplementary Material) (Figure 3A). In contrast, Herceptin had no effect on NK cell migration when cocultured with HER2^−^ cells (data not shown). Interestingly, NK cells in the blank group had a round shape during the 2-h observation in contrast to the Herceptin-treated group, and they were continuously transforming while moving toward the target cells (Figure 3A). By analyzing the number of NK cells that adhered to breast cancer cells, we found that Herceptin enhanced the adhesion of Herceptin-treated NK cells with HER2^+^ cells but not HER2^−^ cells, within 20 min of coculture (Figure 3B). The migration and adhesion of IgG1-stimulated NK cells were increased compared with the blank group and reduced compared with Herceptin-treated cells (Video S3 in Supplementary Material). When cultured alone, NK cells exhibited little change (Video S4 in Supplementary Material). Therefore, Herceptin significantly enhanced the migration and adhesion of NK cells toward HER2^+^ breast cancer cells.

### Herceptin Does Not Affect NKp30, NKG2D, and DNAM-1 Surface Receptors on NK Cells

**Figure 4:**
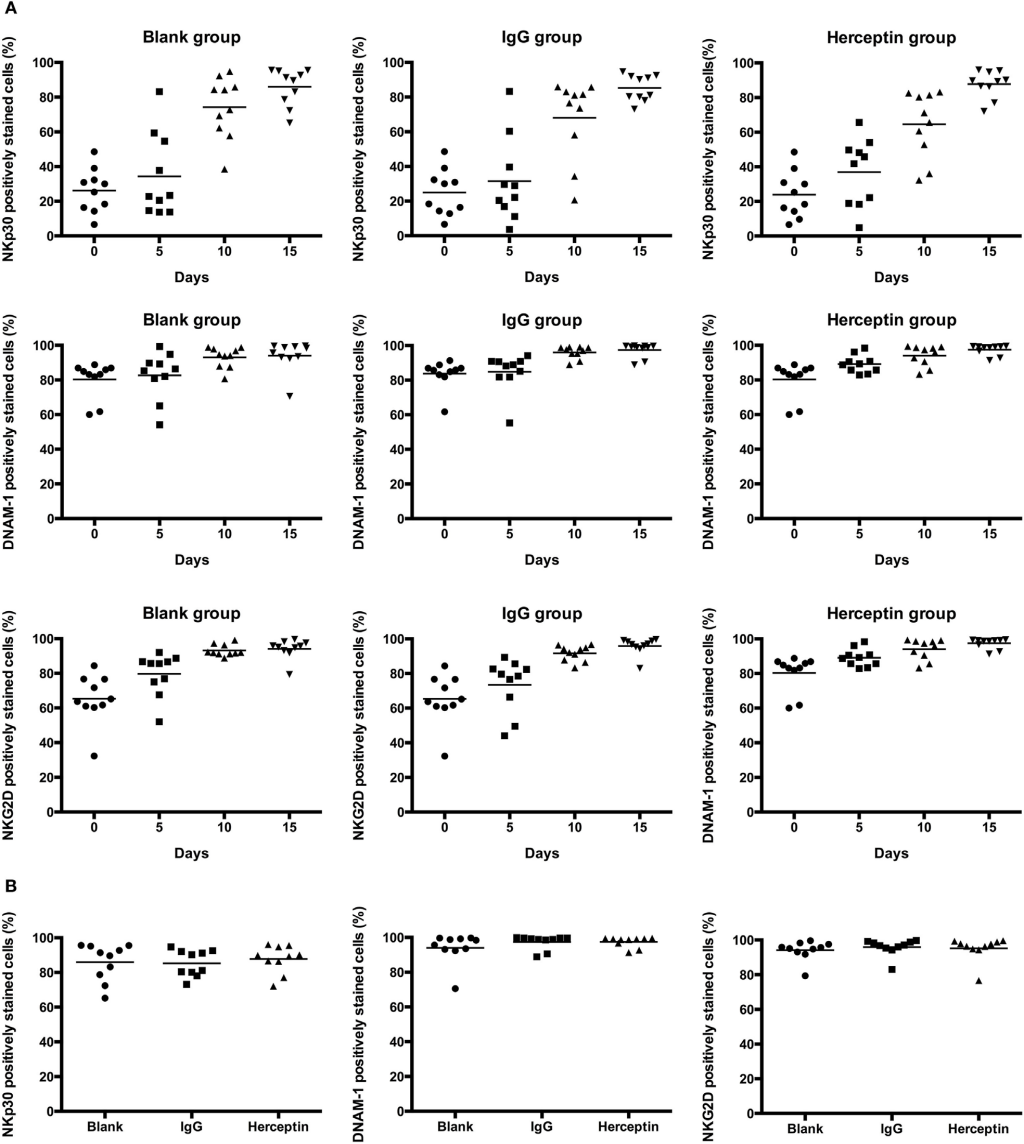
Expression of the cytotoxicity-associated activated receptors, including NKp30, NKG2D, and DNAM-1, in NK cell subsets among the final expansion products cultured in three different environments. Expression in electronically gated CD3^−^CD56^+^ NK cells was analyzed by FCM. **(A)** Changes in the expression of each receptor over 15 days. **(B)** The expression of each receptor on the final expanded NK cells among three groups is shown. The results are presented as the means of 10 patients and error bars represent the SD.

To understand how Herceptin stimulates the activity of NK cells, including proliferation, cytotoxicity, and migration, we evaluated the effects of Herceptin on cytotoxicity-associated activated receptors NKp30, NKG2D, and DNAM-1. Every 5 days, the same number of cells from three groups was analyzed using FCM to examine the expression levels of these receptors. The percentage of receptors means the number of receptors every one hundred NK cells expressing. The number of NK cells expressing these surface activating receptors changed in a similar manner among the blank, IgG1, and Herceptin groups during the 15-day culture period (Figure 4A). The numbers of NK cells expressing these surface activating receptors were increased in all three groups 15 days after culture, but no difference was observed among these three groups (Figure 4B). In addition, the mean fluorescent intensity values, which evaluate the number of receptors on one NK cells, showed the same trend as the frequency in these three groups (data not shown). Therefore, neither IgG1 nor Herceptin influenced the activation of these surface receptors in NK cells.

### Herceptin Interacts with CD16 for Increasing NK Cell Activity

**Figure 5:**
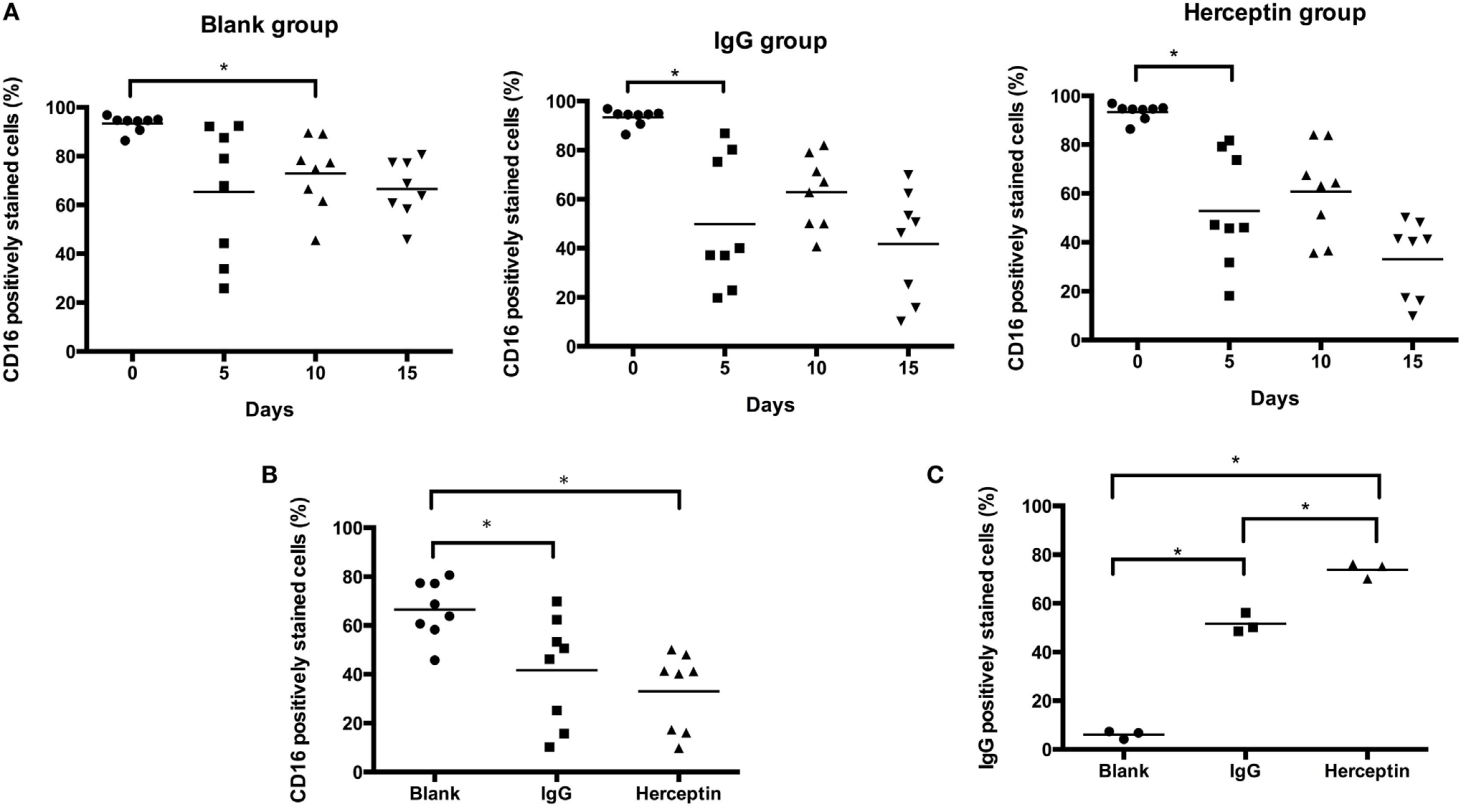
CD16 and Herceptin levels on expanded natural killer (NK) cells among the final expansion products cultured under three different conditions. Expression in gated CD3^−^CD56^+^ NK cells was analyzed by flow cytometry (FCM). **(A)** Changes in the expression of CD16 at 0, 5, 10, and 15 days. **(B)** Comparison of CD16 expression at 15 days between the final expanded NK cells in each of the three groups. **(C)** Amounts of Herceptin and IgG1 remaining on the NK cell surface at Day 15. We used a secondary antibody to detect Herceptin and IgG1 on the NK cells in the final products using FCM. The results shown in **(A,B)** are presented as the means of eight patients and the results shown in **(C)** are presented as the means of three patients. Error bars represent the SD. *p < 0.05.

Ligation of CD16 leads to activation of this receptor and activation of CD16 regulates NK cell activity. Therefore, the unbinding-CD16 expression level represents the activation status after CD16 ligation. Next, we determined the effects of Herceptin on CD16 activation in NK cells by detecting unbinding-CD16 expression level in NK cells. We examined the percentage of CD16 positively stained cells in CD3^−^CD56^+^ NK cells by using FCM analysis (representative FCM plots are shown in Figure S2 in Supplementary Material). Although both Herceptin and IgG1 induced decreases in the number of NK cells expressing unbinding-CD16 starting at 5 days after culture, compared to the blank group, Herceptin treatment had a more potent effect (Figures 5A,B).

We also examined the interaction between antibodies and NK cells during short-term culture. There was a more rapid reduction in binding of anti CD16 antibody after Herceptin incubation compared to control IgG1 (Figure S3 in Supplementary Material, representative FCM plots were shown in Figure S4 in Supplementary Material). The result thus suggested that CD16 bind more efficiently to Herceptin than to control IgG1 and was in agreement with the results in Figure 5B.

We further examined the amount of IgG1 and Herceptin binding to CD16 on the cell surface of NK cells by staining using an anti-IgG antibody and FCM analysis. Herceptin was present on the NK cells and occupied CD16 sites (73.80 ± 3.24%), which were higher than that in the IgG1-treated group (51.60 ± 3.99%) at 15 days after culture (Figure 5C).

Our data suggested that Herceptin induced more CD16 occupation, as compared to IgG1, indicating that Herceptin has a higher effect on CD16 activation. Compared to IgG1, the interaction between Herceptin and CD16 might induce stronger CD16 activation, which may help activate a series of signaling pathways downstream from CD16 to stimulate cell cytotoxicity and migration.

### The Clinical Efficacy of Herceptin-Treated NK Cells for a Patient with Breast Cancer Expressing HER2^+^—A Pilot Trial

**Figure 6:**
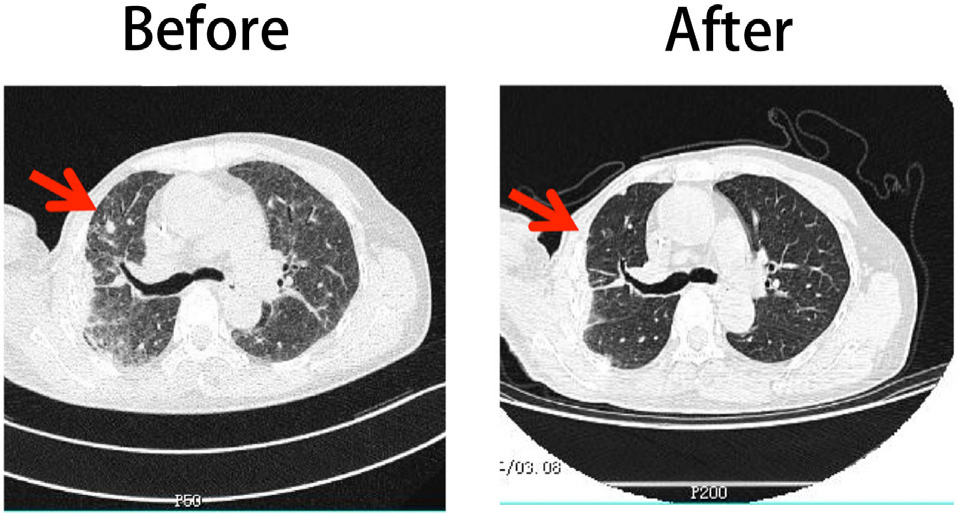
Comparison of the patient’s CT scan images before and after Herceptin-treated natural killer (NK) cell therapy. The left column shows the image obtained before treatment and the right column shows the image obtained after four cycles of NK cell therapy.

A 67-year-old female patient was diagnosed with HER2-expressing breast cancer 13 years prior. She underwent right radical mastectomy and partial left mastectomy followed by chemotherapy. She did not receive complete Herceptin treatment because of severe cardiotoxicity. She received a combination treatment, including chemotherapy, radiotherapy, endocrine therapy, and biotherapy during the previous 10 years. Six months prior to the Herceptin-treated NK cell treatment, the patient had a pulmonary metastasis based on B-mode ultrasound and computed tomography examination and then received Lapatinib-based chemotherapy treatment. Unfortunately, none of the therapy could stop the cancer progression. The shadows and nodules continued to grow. The Herceptin-NK cell treatment was approved by the ethical committee of Tianjin Medical University Cancer Hospital with written consent. All the procedures of NK cell expansion were operated in strictly controlled GMP lab and all reagents used in the procedure met the requirement of China Food and Drug Administration. No FCS was used for the preparation of adoptively transferred cells. Before the treatment, the biological safety and efficiency was guaranteed by a series QC steps of bacterial detection, toxin detection, immunotyping, and cytotoxicity examination. The patient underwent four cycles of Herceptin-treated NK cell treatment at intervals of 2 months. 5.9 × 10^9^, 3.9 × 10^9^, 8.1 × 10^9^, and 6.5 × 10^9^ Herceptin-treated NK cells were administered at each occasion. The treatment was well tolerated, with no treatment-related morbidity, life-threatening complications, or side effects. The diameter of the foci before the first treatment was approximately 36.0 mm and decreased to 12.0 mm after four cycles of Herceptin-treated NK cell treatment (Figure 6), which was considered a partial response. This evidence supports the application of Herceptin-treated NK cells as a potential clinical treatment.

## Discussion

The functional status of the host immune system affects the efficiency of chemotherapy and radiotherapy (25). ACT improves the host immune functions to efficiently treat cancer. Several types of immune cells have been used to enhance the immune activities, such as NK cells, cytokine-induced killer cells, lymphokine activated killer cells, γδT cells, αβT cells, cytotoxic T cells, dendritic cells, and chimeric antigen receptor (CAR)-redirected T cells. NK cells are characterized by direct and efficient main histocompatibility complex-unrestricted antitumor activity. This advantage makes NK cells a proper candidate for ACT. However, NK cell function is reported to be impaired in cancer patients (26). Therefore, we developed an *ex vivo* NK cell expansion approach to acquire fully functional NK cells for NK cell-based ACT.

Compared with CAR-redirected T cells, the main disadvantage of NK cells is that their cytotoxicity is not specific for target cells. The generation of CAR-modified T cells that specifically kill the targets involves genetic manipulation of donor lymphocytes, requires specialized facilities for generation and is expensive compared with NK cell expansion. CAR-modified NK cell are a new tool for the rapid development of adoptive immunotherapy for tumors that has been reported in recent years (27). CAR-modified NK cells have targeted cytotoxic activity, overcome the tumor immunosuppressive microenvironment and disrupt the host immune tolerance. However, CAR-NK cells do not expand in response to an applied stimulus that is in contrast to current CAR-T cells (28). Therefore, the CAR-driven expansion observed for CAR-T cells seems to be unlikely to occur in NK cells, limiting their long-term antitumor effects. Using the low-cost method reported in the present study, NK cells with specific targeting were easily amplified for use in ACT.

Herceptin has limited effectiveness when used alone (3). The percentage of HER2 expressing patients who responded to Herceptin was reported to reach merely 30% (29). On the other hand, the majority of patients who achieve an initial response to Herceptin-based regimens generally acquire resistance within one year (30). Herceptin is also known to exert specific side effects on the heart when used in combination with chemotherapy (31). In the present study, using antibody-dependent cell-mediated cytotoxicity (ADCC) with Herceptin, NK cells had targeting ability. Therefore, the aforementioned limitations of Herceptin treatment were overcome.

Human NK cells have two distinct subsets identified by the cell surface density of CD56; approximately 90% are CD56^dim^ cells and 10% are CD56^bright^ cells (32). CD56^dim^ NK cells exhibit a marked cytotoxicity potential, while CD56^bright^ cells have a greater capacity to secrete cytokines (33). Peripheral blood CD56^bright^ NK cells lack CD16 expression (34), and CD56^dim^ NK cells have a higher level of CD16. Our results showed that cytokines amplified the number of CD56^bright^ NK cells; however, Herceptin could expand the proportion of CD56^dim^ NK cells. CD16 expression level was lower in the Herceptin-treated NK cells that have a higher level of CD16, indicating that more CD16 on NK cells was ligated and occupied by Herceptin in Herceptin group and Herceptin might induce NK cells to exert a high level of cytotoxicity.

NKp30, NKG2D, and DNAM-1 are important surface activated receptors involved in NK cell cytotoxicity. Almost all mature NK cells express NKp30 (35). NKG2D combined with specific ligands transmits signals required for cytotoxicity through DAP10 and DAP12 (36). DNAM-1 combined with the specific ligand would cause tighter adhesion between NK cells and target cells (37). In this experiment, we compared the expression level of the receptors in two different aspects. We compared the receptor expression level on Days 0, 5, 10, and 15 in each group. In all three groups, receptors expression levels were increased during 15-day culture with enhanced adhesion and cytotoxicity. We inferred that increased NK activity during culture period was due to the cytokines induced the increasing expression of activation receptors that is in accordance with previous study. It was reported that cytokines, such as IL-2 and IL-15, could increase the expression level of NK cells surface activated receptors during *ex vivo* expression (38). Moreover, in a clinic trial, the IL-2 treatment resulted in expansion of NK cells in the blood along with increased NK cell expression of NKp30 (39). On the other hand, when compared receptor expression levels among three groups on Day 15, we found that there was no significant difference among three groups. But the expansion, adhesion and cytotoxicity ability among three groups on Day 15, we found significant increase in Herceptin group and IgG1 group than control group. Therefore, we speculated that increased NK activity on Day 15 among three groups is not related to the increasing expression level of activation receptors and Herceptin did not significantly increase the number of activation receptors in relation to the control.

Most clinical studies have used *ex vivo* activated NK cells instead of *in vivo* activated NK cells (40). The *ex vivo* activated NK cells express low or undetectable levels of CD16; therefore, they are not a choice for ADCC (40). However, the interaction between Herceptin and CD16 induced longer and stronger CD16 occupation than IgG1 in the present study, and Herceptin-treated NK cells were only effective against HER2^+^ breast cancer cells. The hypothesis that ligation of CD16 by Herceptin induces NK cell hyper-responsiveness is strengthened by the finding that IgG1 had no effect on the cytotoxicity toward target cells. The Herceptin-occupied CD16 sites remaining on the NK cells maintained the activity of NK cells against target cells. The Fc region of Herceptin was ligated with CD16 on NK cells and Fab regions related to HER2 on target cells, while the Fab regions of IgG1 did not recognize HER2 on the target cells to mediate ADCC. We hypothesized that other monoantibodies, such as Cetuximab, may have the same effect on colorectal cancer cells.

CD16, occupied by antibodies, is coupled to ITAM-containing signaling adaptors (41) and then activates a downstream signaling pathway. The important relevant proteins in the downstream pathways include PI3-K, VAV1, and RAC (42). RAC induces granule secretion and cytotoxicity (43). PI3-K correlates with cell proliferation (44). VAV1 is an important factor involved in cell transformation and migration (45). These proteins were associated with proliferation, migration, and cytotoxicity. More research will be performed to study the mechanism underlying the correlations between these signaling pathways and CD16 to further explain our hypotheses. Notably, CD16 internalization may affect the decrease in CD16 accessibility. However, in this article, we mainly considered the interaction between Herceptin and accessible CD16 on NK cells. We will evaluate the level of CD16 internalization and how the internalized CD16-Herceptin complex transduces or regulates signaling in NK cells.

In previous studies, Herceptin was used as a target therapy to directly inhibit the growth of HER2-positive cancers by blocking the transduction of HER2-mediated growth signal in cancer cells. Few reports were focused on the antitumor effects of Herceptin-mediated immunotherapy. Until now we have not find similar *in vitro* method to observe the effect of Herceptin on NK cells. Though ADCC effect was reported to induce tumor inhibition both *in vitro* and *in vivo*, no relationship between Herceptin and lymphocytes activation has been identified except ref.20. It is a clinic dilemma that high-dose Herceptin could induce resistance with high probability but low-dose Herceptin could not get the ideal clinic benefits. In this study, we proposed a novel Herceptin-mediated NK activation method to amplify NK cells with considerably lower dose of Herceptin pretreatment *in vitro* which displayed significantly enhanced cytotoxicity against HER2-positive breast cancer cells and proposed an alternative solution for cancer patients resistant to conventional Herceptin treatment. Furthermore, different from previous study, we observed that Herceptin could increase NK cells amplification and activation at the expansion stage rather than effect stage, which indicated that an alternative mechanism except ADCC effect has involved in Herceptin-mediated NK activation. During expansion stage, CD16 are occupied which was bound by Herceptin to activate downstream signaling pathways, such as RAC, VAV, and ERK. CD16-mediated activation of the downstream signaling pathways is related to enhanced adhesion and cytotoxicity of NK cells at the expansion stage while the ADCC effect further increases the cytotoxicity of NK cells at the effect stage when target cells expressed specific HER2 protein on the surface. Therefore, this research proposed an alternative Herceptin-mediated immunotherapy for HER2-positive cancer and optimized the method of NK cell expansion for future clinical application.

Since Herceptin’s irreversible cardiotoxicity, this study found a new alternative way to use Herceptin in HER2-positive patient who has poor cardiac function. For patients with Herceptin resistance, using Herceptin-induced NK cell therapy might provide an alternative treatment to overcome the problem of drug resistance. In this study, we specifically described and analyzed the Herceptin’s effect on NK cell expansion, activation, adhesion, and cytotoxicity and confirmed that Herceptin-mediated NK activation is feasible and repeatable among different cancer patients no matter the disparity of age, gender, clinical stage, and response to conventional therapy. Therefore, we developed an easy, stable, accurate method with high repeatability in this study and could be feasible in clinic application. Since less amount of Herceptin was used in NK cells amplification, the culture cost of Herceptin-NK cells is comparably lower than standard Herceptin therapy and would be an economic way in breast cancer treatment. However, since no animal models were established in this study, it is difficult to evaluate the ability of adoptively transferred cells to traffic to tumor sites and to enter into cancer tissues. We would like to improve our study in the future.

Herceptin and the isotype antibody belong to the same allotype. One interesting finding from our study is that Herceptin has a higher ability to bind to CD16 on NK cells and exerts more potent effects on NK cell proliferation compared to IgG1 treatment. Glycans have many structural and functional roles in antibodies, and the effector function can be altered by different glycoforms in antibodies (46). It has been shown that the immunogenicity, pharmacokinetics, distribution, solubility, and stability of the antibodies could be affected by glycosylation in the Fc fragment (47). Glycosylation on the IgG Fc fragment mediates the enhancement of the functions of Fc receptors (48). One of the important glycan patterns in the Fc fragment is *N*-acetylglucosamine. Studies have shown that the Fc fragment with the low level of *N*-acetylglucosamine stimulates a higher level of activity compared to that with bisected *N*-acetylglucosamine (49). Another glycan pattern is fucosylation. It was reported that a low level of Fc fragment core-fucosylation enhances several biological functions of the Fc fragment (50).

IgG1 and Herceptin have the same N-linked glycosylation site at the position of Asn297 in the Fc region of each heavy chain (51). It has been reported that the cell culture condition, fermentation method, and other factors can alter the glycosylation of proteins (52). Herceptin was expressed in CHO cells and IgG1 used in this study was purified from human plasma. Since control IgG1 is of different origin, as compared to Herceptin, they might bind and cross-link Fc receptors with a significantly lower effectiveness. It is possible that the glycosylation status between Herceptin and IgG1 differs. We presumed that the differences in glycosylation pattern on the Fc fragment might contribute to differences in the binding capacity to CD16 in NK cells by Herceptin and IgG1. Therefore, Herceptin and IgG1 have different effects on NK cells. As shown in Figure 5C, NK cells are largely Herceptin positive at the end of the incubation period, thus suggesting that, maybe, half-life of control IgG1 is also significantly shorter. In addition, the IgG1 used for this study was purchased as a lyophilized protein. IgG1 may become multimeric aggregated and lose functions under the reconstituted condition (53). Therefore, reconstituted IgG1 might not be as active as Herceptin for stimulating CD16 in NK cells. In this experiment, we inferred that Herceptin occupying CD16 (Fc receptor) on NK cells increase their effects. In the further experiments, we could focus on CD16 and Fc region to evaluate the CD16 functions on NK cells.

For patients, Herceptin is a heavy economic burden. Combining Herceptin and NK cells together is an economic way in breast cancer treatment. Even though considering the cost of culture medium, the total expense is very low compared to directly use Herceptin as targeted medicine administrated passively. However, we just observed the effect of Herceptin on NK cells and we will ran more animal experiments to evaluate the ability of Herceptin-treated NK cells transferring to tumor sites *in vitro* next step.

## Conclusion

Our results demonstrated that Herceptin promoted NK cell proliferation, cytotoxicity, and migration by occupying CD16 to enhance the antitumor effects of NK cells on HER2^+^ breast cancer cells. We initially developed an easy, stable, accurate method with high repeatability and could be feasible in clinic application. This transitional research uses Herceptin to optimize the original way of NK cell expansion. The clinical efficacy of Herceptin-treated NK cells for a patient with HER2^+^ and Herceptin-intolerant breast cancer was identified in a pilot case. Therefore, the use of Herceptin-treated NK cells might serve as a promising alternative treatment for patients with HER2^+^ and Herceptin-intolerant breast cancer.

## Ethics Statement

This study was carried out in accordance with the recommendations of the ethical standards of the Institutional Review Committee on Human Research of the Tianjin Medical University Cancer Institute and Hospital with written informed consent from all subjects. All subjects gave written informed consent in accordance with the Declaration of Helsinki. The protocol was approved by the Institutional Review Committee on Human Research of the Tianjin Medical University Cancer Institute and Hospital.

## Author Contributions

Conception and design; drafting of the manuscript: XT, JY, and XR. Execution of experiments: XT, LW, WY, and NZ. Acquisition of data: XT, WY, and LW. Analysis and interpretation of data: XT, FW, JY, and XR. Pilot trial: YH and XZ. Obtained funding: XZ. Critical revision of the manuscript; final approval of the version to be published, study supervision; and agreement to be accountable for all aspects of the work: JY and XR.

## Conflict of Interest Statement

The authors declare that the research was conducted in the absence of any commercial or financial relationships that could be construed as a potential conflict of interest.

## Supplementary Material

Video S1 Herceptin-treated NK cells co-cultured with SK-BR-3 cells.

Video S2 NK cells in Blank group co-cultured with SK-BR-3 cells.

Video S3 IgG1-treated NK cells co-cultured with SK-BR-3 cells.

Video S4 Herceptin treated NK cells cultured alone.

## Abbreviations

ACT, adoptive cell therapy; ADCC, antibody-dependent cell-mediated cytotoxicity; CAR, chimeric antigen receptor; ELISA, enzyme-linked immunosorbent assay; FCM, flow cytometry; HER, human epidermal growth factor receptor; IFN, interferon; IL, interleukin; LDH, lactate dehydrogenase; NK, cell natural killer cell; PBMC, peripheral blood mononuclear cell; PBS, phosphate-buffered saline; siRNA, small-interfering RNA.
