# Supplementary material for: Herceptin Enhances the Antitumor Effect of Natural Killer Cells on Breast Cancer Cells Expressing Human Epidermal Growth Factor Receptor-2
Source: Front Immunol. 2017 Oct 30;8:1426. doi: 10.3389/fimmu.2017.01426 (PMC5670328; doi:10.3389/fimmu.2017.01426)
Supplement: Supplementary file 5 [file Image_1.PDF]

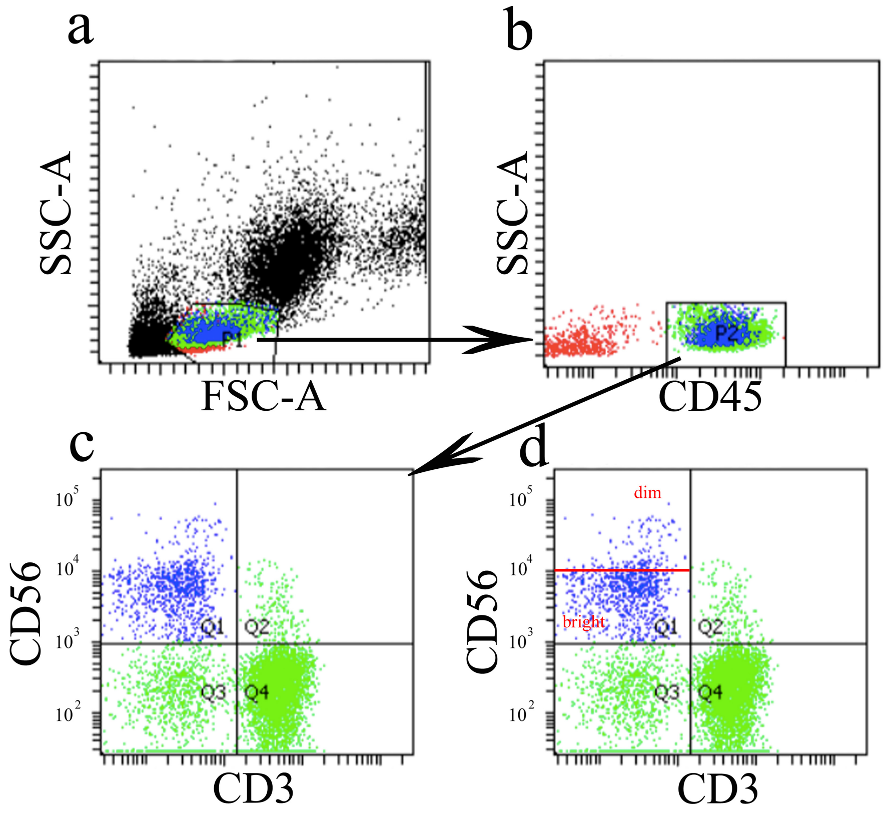

Supplementary figure 1 Gating strategy employed for the percentages of CD56^{dim} and CD56^{bright} NK cells Representative flow dot plots from one donor showing the gating strategy for the percentages of CD56^{dim} and CD56^{bright} NK cells. Sequentially: **a** cells were gated using SSC-A and FSC-A **b** CD45 was used to gate out monocytes **c** CD3 and CD56 were used to select NK cells **d** 10^4 was used to determine the percentages of CD56^{dim} and CD56^{bright} NK cells

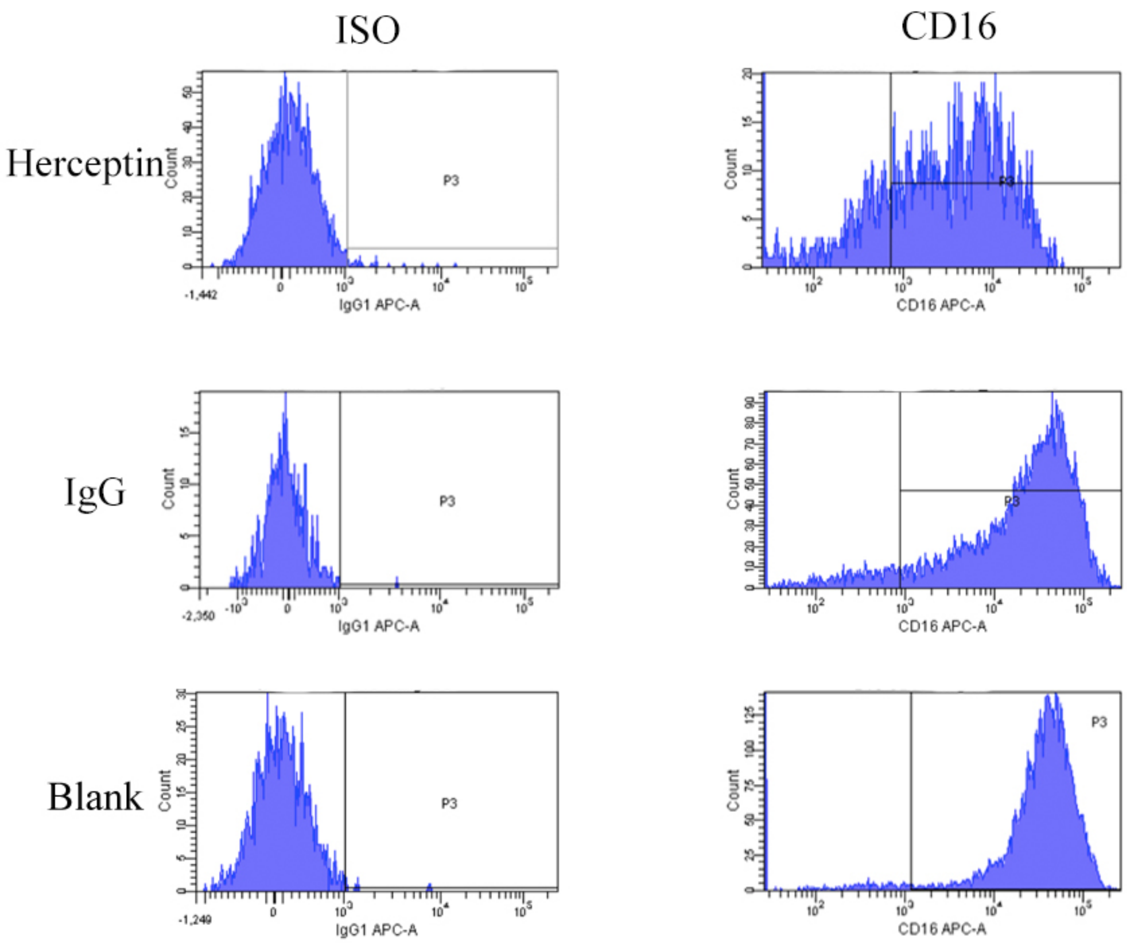

Supplementary figure 2 Representative plots for the different groups of NK cells at Day 15
Expression in gated $CD3^{-}CD56^{+}$ NK cells was analyzed by FCM.

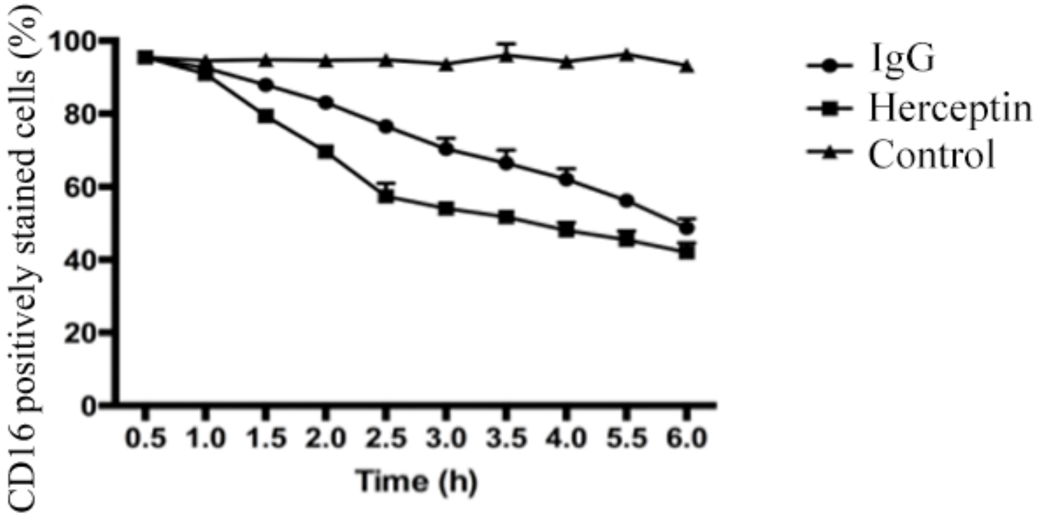

Supplementary figure 3 Interaction between Herceptin or IgG and NK cells NK cells were co-cultured for the indicated times with Herceptin or IgG, expression in gated $CD3^{-}CD56^{+}$ NK cells was analyzed by FCM. We use percentage of CD16 positively stained cells to evaluate the antibody's binding ability over time.

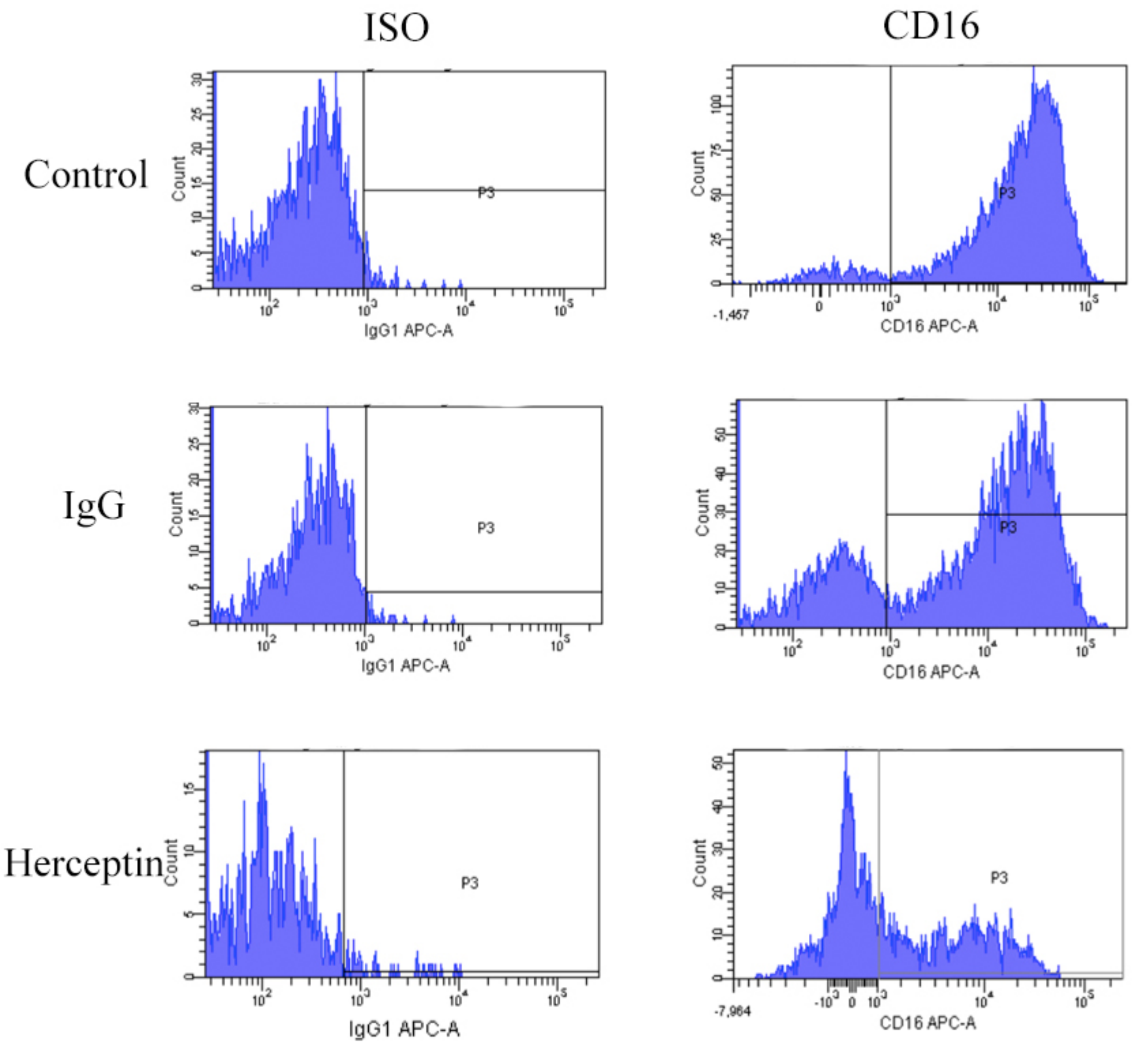

Supplementary figure 4 Representative plots at 4.0h in Supplementary figure 3
